# Structural interrogation of phosphoproteome identified by mass spectrometry reveals allowed and disallowed regions of phosphoconformation

**DOI:** 10.1186/1472-6807-14-9

**Published:** 2014-03-11

**Authors:** Arun Kumar Somavarapu, Satish Balakrishnan, Amit Kumar Singh Gautam, David S Palmer, Prasanna Venkatraman

**Affiliations:** 1 Protein Interactome Lab for Structural and Functional Biology, Advanced Center for Treatment Research and Education in Cancer, Tata Memorial Centre, Kharghar, Navi Mumbai, Maharashtra 410210, India; 2 Department of Pure and Applied Chemistry, University of Strathclyde, Thomas Graham Building, 295 Cathedral Street, Glasgow G1 1XL, United Kingdom

**Keywords:** Phosphorylation, Mass spectrometry, Structure, Dynamics, Accessibility, Bioinformatics

## Abstract

**Background:**

High-throughput mass spectrometric (HT-MS) study is the method of choice for monitoring global changes in proteome. Data derived from these studies are meant for further validation and experimentation to discover novel biological insights. Here we evaluate use of relative solvent accessible surface area (rSASA) and DEPTH as indices to assess experimentally determined phosphorylation events deposited in PhosphoSitePlus.

**Results:**

Based on accessibility, we map these identifications on allowed (accessible) or disallowed (inaccessible) regions of phosphoconformation. Surprisingly a striking number of HT-MS/MS derived events (1461/5947 sites or 24.6%) are present in the disallowed region of conformation. By considering protein dynamics, autophosphorylation events and/or the sequence specificity of kinases, 13.8% of these phosphosites can be moved to the allowed region of conformation. We also demonstrate that rSASA values can be used to increase the confidence of identification of phosphorylation sites within an ambiguous MS dataset.

**Conclusion:**

While MS is a stand-alone technique for the identification of vast majority of phosphorylation events, identifications within disallowed region of conformation will benefit from techniques that independently probe for phosphorylation and protein dynamics. Our studies also imply that trapping alternate protein conformations may be a viable alternative to the design of inhibitors against mutation prone drug resistance kinases.

## Background

Phosphorylation is a reversible post translational modification of proteins that regulates many vital process like cell cycle, cell proliferation, signal transduction and cell death to name a few [1-3]. It is also a fundamental mechanism by which a message from a small set of genes is translated into pathway based spatio-temporal regulation of cellular function [4-6]. At least 1/3 ^rd^ of cellular proteins are estimated to be phosphorylated often at more than one site [7,8]. Therefore the need for characterizing global changes in phosphorylation cannot be overemphasized and it is a basic requirement for understanding functional biology at systems level [5,9-11]. By such criteria these studies are not an end but the beginning of the way in which science will be conducted and interpreted increasingly in the future. Multiple techniques and knowledge from several disciplines like biochemistry, biophysics, structural and cell biology, computational and bioinformatic studies will be necessary for integrated and comprehensive understanding of biology and its intervention.

With the advent of fast high resolution liquid chromatography (LC) techniques, identification of global changes in the proteome such as post-translational modifications by LC-MS has become the method of choice [10,12-15]. Identification of phosphorylation by HT-LC-MS is primarily dependent on the use of accurate mass of the peptide, sequence from tandem MS and a search for its match within a theoretically generated database of proteins [5,12,13]. Assignment relies on probability scores [16,17]. Until recently, probable errors within HT-MS data could only be verified manually [18]. Errors in such large scale identifications have been considerably reduced by technological advancements that ensure high accuracy of identification, enrichment of phosphorylated proteins using metal affinity columns, quantifying relative proportions of the phosphorylated and unphosphorylated species and integration of new bioinformatic methods and algorithms into search engines [15,18-24]. Despite all these approaches and stringent rules imposed by the investigators, like all other HT studies, some false positive and false negative identification of phosphorylation sites is inevitable. As the amount of information on proteome wide phosphorylation is being gathered at a rapid rate, validation of these identifications remains a concern and a challenge. Besides it is expected for the future of mass spectrometry that the technique stands validated on its own.

To address this issue we chose to apply some of the fundamental structural rules to a vast depository of MS derived data, PhosphoSitePlus, on protein phosphorylation. We simply asked how many of the identified phosphosites are in compliance with a major structural rule, that is, degree of solvent accessibility. Accessibility of phosphorylation sites in proteins and conformational changes induced by phosphorylation have been addressed before [25-27]. Phosphorylation has been detected mostly in flexible, disordered [28] and in accessible regions of the protein [29]. Few elegant studies have described the structural basis of phosphorylation [25,30,31]. Conformational changes in proteins before and after phosphorylation [25,26] have been demonstrated using crystal structures of the same protein in its unphosphorylated and phosphorylated forms. Yet to the best of our knowledge there is no comprehensive analysis of structures of a large body of MS derived phosphosites and its application for subjective validation of experimentally determined phosphosites. Information from structural analysis would be unbiased as it is blind to the source of data and more importantly it is blind to the technique of MS employed. Since solvent or surface accessibility of a sequence is one of the fundamental requirements for a kinase mediated phosphorylation, we asked the following: to what extent can the existing structural information help to identify *genuine* phosphorylation sites?

## Results and discussion

### Analysis of the phosphorylated sequences from the PhosphoSitePlus

One of the primary requirements for a site to get phosphorylated is its accessibility to a kinase, a parameter, that can be calculated using Solvent Accessible Surface Area or SASA of a sequence for which structural information is available. Phosphosequences from PhosphoSitePlus were downloaded, matched with the PDB data base and coordinates were used for calculating rSASA using Parameter Optimized Surfaces in the stand alone mode. For the a few proteins (2.3%) the matched structures were of the phosphorylated sequence but for majority of others (97.7%) they were of non phosphorylated forms. SASA values and rSASA values were extracted in the context of the octapeptide where phosphorylated residue occupies the 4^th^ position. SASA value has been previously used to evaluate phosphorylation events in mitotic check point proteins [29], by us to identify novel substrates of endoproteases [32] and by the Craig and Sali group for the identification of Granzyme substrates [33]. Out of 16,528 unique phosphorylation sites in the phosphosite database 3579 sites were present in the disordered region (no co-ordinates) and 315 sites were present at the extreme termini (Additional file 1: Figure S1 and Table S1). Phosphorylation at these sites by a kinase is highly likely and thus stands validated by criteria of accessibility. For other sites where co-ordinates were available for the octapeptide sequence (please see methods), only protein structures which covered 70% of the primary sequence were considered. This stringency narrowed down the study to 5947 sites which were further analyzed using a reference data set of proteins created from Protein Data Base (PDB) with solved crystal structure of phosphorylated residues (Additional file 1: Table S2).

### Comparative analysis of PDB and Phosphosite

**Figure 1:**
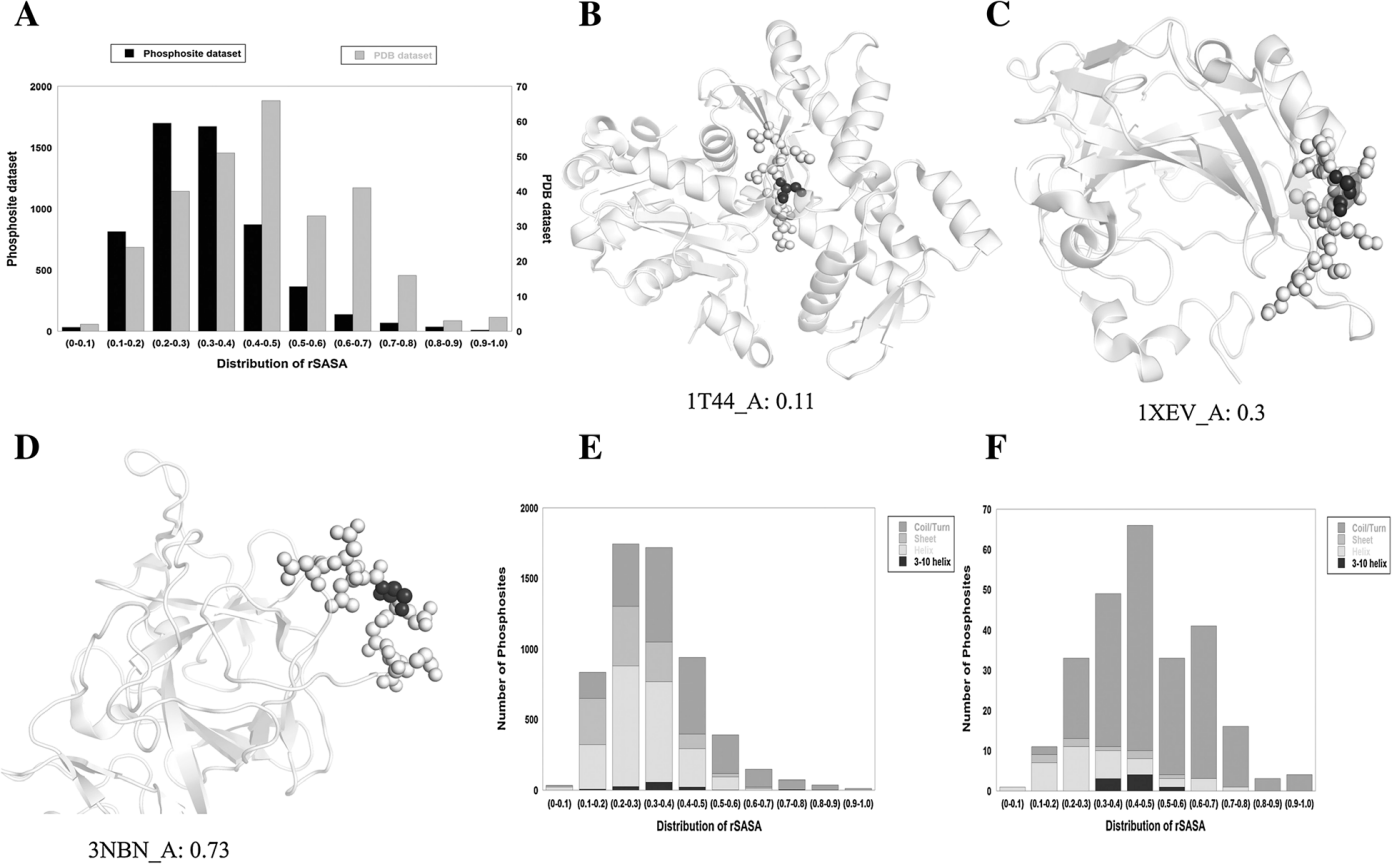
**Comparative analysis of PDB and Phosphosite-plus datasets. A)** rSASA values from Phosphosite-plus and PDB datasets were binned at regular intervals with a difference of 0.1. Data from phosphosite-plus were plotted on Y1 axis and those from PDB were plotted on Y2 axis. Majority of phosphorylation sites in PDB dataset are in well accessible regions of the protein while in PhosphoSitePlus, they are found in moderately accessible regions. Representative structures where different phosphosites are found in three different regions of accessibility are shown. **B)** Actin protein (PDBID: 1 T44) where the site lies in inaccessible region (rSASA: 0.11), in **C**, carbonic anhydrase II (PDBID: 1XEV) the site is in a moderately accessible region (rSASA: 0.3) and in **D**, recombining binding protein suppressor of hairless (PDBID: 3NBN), in a well accessible region (0.73). All protein structures were fetched from PDB by matching the Uniprot ID of the protein from the phosphosite data. Distribution of octapeptide secondary structure and their accessibility. **E)** Octapeptides from Phosphosite-plus dataset and **F)** Octapeptides from the PDB dataset.

In the PDB, 282 unique phosphorylation sites were found within prokaryotic, eukaryotic, bacterial and viral proteins (Additional file 1: Table S2). In these proteins besides Ser/Thr/Tyr (conventional) residues Asp/His/Cys residues (unconventional) were also phosphorylated. Conventional and unconventional phosphorylation sites from Pro and eukaryotic proteins were independently segregated. Conventional phosphorylation of the eukaryotic proteins from the PDB database and PhosphoSitePlus were then compared. Data were binned in blocks of 0.1 rSASA units (0–0.1, 0.1-0.2 etc. upto 0.9-1.0). The mode for the PDB data lies in the range of 0.4-0.5 and for phosphosite it is in the range of 0.2-0.3 (Figure 1A). The median for the PDB data is 0.42 and for phosphosite it is centered on 0.3.

While most (58.4%) of the experimentally determined phosphorylation sites occur in moderately accessible (0.2-0.4) regions of proteins, the PDB is marked by (54.47%) phosphorylated residues in more accessible regions (0.4-0.7). This distribution was verified after energy minimization of the structures and the results remain the same (Additional file 1: Figure S2). Representative protein structures in which the phosphosite lies in this range of rSASA values are shown in Figure 1B,C,D. In protein Actin (PDB 1T44), the site is in an inaccessible region (0.11), while in carbonic anhydrase II (PDB 1XEV), the site is in a moderately accessible (0.3) region and the phosphosite in recombining binding protein suppressor of hairless (PDB 3NBN), is in a well accessible region (0.73).

Even within the PDB, number of sites within the highly accessible region (>0.7) is rather small. Remarkably a significant amount of phosphorylation events occur in relatively inaccessible regions of the protein (rSASA < 0.2). PDB protein structures are of phosphorylated forms. Therefore this inaccessibility in some cases may be due to the conformational changes induced by phosphorylation. Sequestration of a phosphorylation site could be an evolutionary strategy evolved to protect these sites from indiscriminate or untimely dephosphorylation. PhosphoSitePlus on the other hand is predominantly represented in the PDB in their unphosphorylated forms which may be different from the corresponding phosphorylated structures. Therefore presence of these sequences in relatively less accessible regions of the protein prior to phosphorylation is surprising.

Observed differences in the pattern of distribution of rSASA values between PDB and phosphosite data is also reflected in the secondary structural properties of the phosphorylated sequences. PDB sequences are enriched in turn/coil conformation (79.7%) while phosphosite is populated in helical structures (38.14% helix and 39.7% turn) and to a lesser extent by beta sheets (20.1%) which may explain lower accessibility values (Figure 1E and F). Abundance of helical structures in phosphorylation sites has not been noted before.

### Conformational Map of phosphorylation

**Figure 2:**
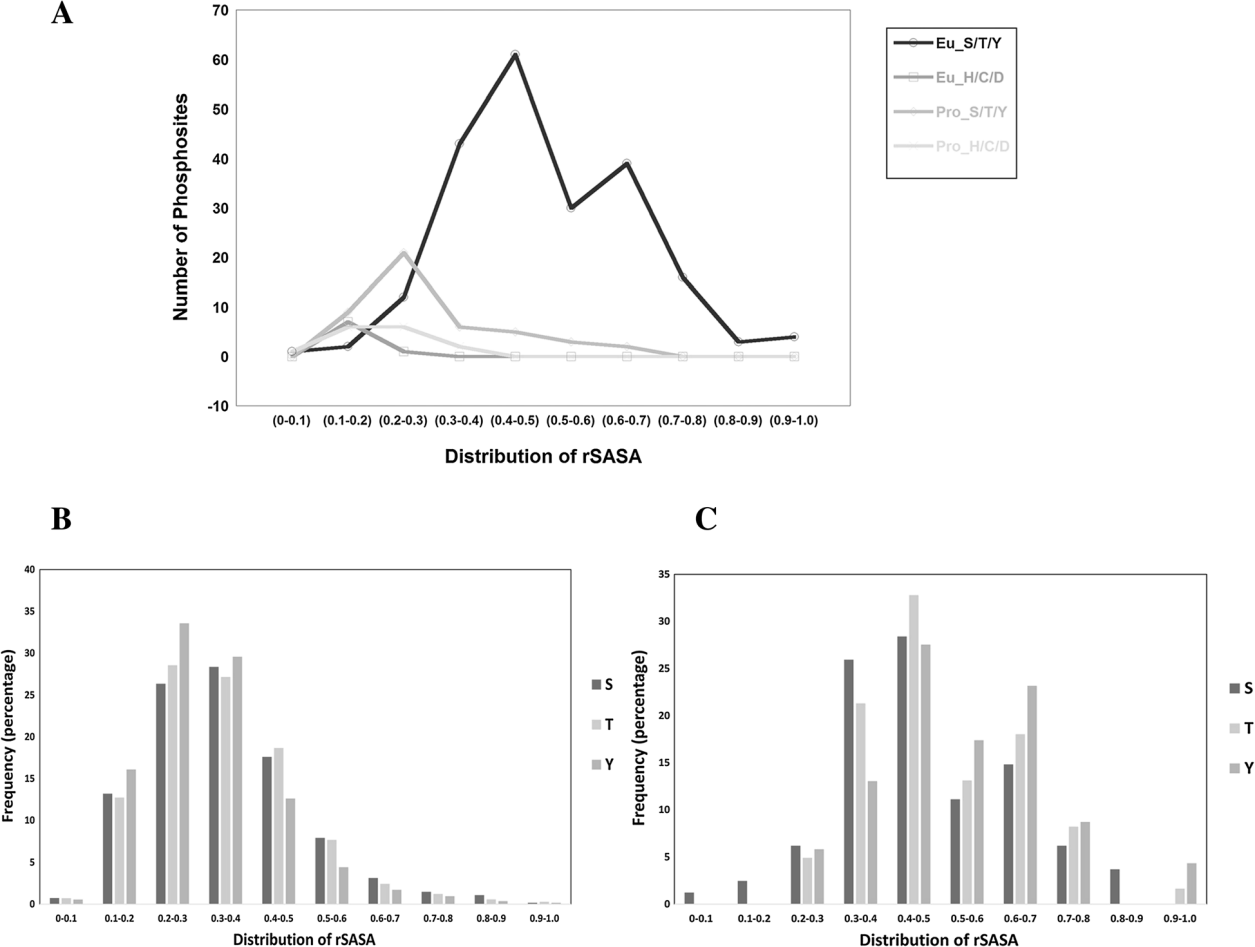
**Conformational map of the phosphoproteome. A)** Phosphorylated proteins in the PDB data set were classified under eukaryotes and prokaryotes. These were further subdivided in to subsets of conventional (Eu_S/T/Y, Pro_S/T/Y) and unconventional (Eu_H/C/D, Pro_H/C/D) phosphorylation sites. All subsets were binned with size interval of 0.1. Majority of eukaryotic conventional phosphorylation sites registered rSASA values >0.2. The rSASA values were also calculated for the single residues Ser, Thr and Tyr from phosphosite data **B)** and from the PDB data **C)**.

To better understand the distribution of rSASA values and to define a lower limit above which a kinase mediated phosphorylation can be considered likely, we analyzed the PDB data set more carefully. rSASA values obtained from conventional and unconventional phosphorylation sites were plotted (Figure 2A). Regardless of their origin rSASA values of unconventional phosphorylation sites were always less than 0.3 (21/23). A significant percentage of conventional phosphorylation sites (7.1%) in the eukaryotes had rSASA values <0.3 indicating lesser accessibility/inaccessibility of these sites. As mentioned before, it is possible that post-phosphorylation these sequences may have moved into the buried region of the protein. However there is no overwhelming evidence to extrapolate this explanation to all such examples.

A careful look at the conventional phosphorylation indicates a bi-modal distribution. After a major peak centered on 0.4-0.5 (Figure 2A), there is decrease in phosphosites within the 0.5-0.6 bin, followed by a small increase in number of proteins with rSASA values between 0.6 and 0.7. We addressed this issue by considering the preferential abundance of Ser, Thr and Tyr phosphorylations. Residue level rSASA values were calculated for the PDB and PhosphoSitePlus data bases (Figure 2B and C). The observed bias is not a reflection of the type of residues that is phosphorylated and the bimodal distribution is consistently seen only with the PDB data base and not the phosphosite data. No plausible explanation seems obvious at this time.

To explain the presence of phosphorylated residues with rSASA values less than 0.3 within the PDB data set, we surveyed the literature (Additional file 1: Table S3). Following facts were observed a) for some of these phosphorylation sites electron density could be assigned to another functional group for example strontium or sulphur, b) phosphorylation was not intended or expected at that site and therefore may not be enzymatic, c) few represent autophosphorylation events in kinases and d) these events represent structural intermediates that were inadvertently captured but the origin of phosphorylation remains a mystery. For example for PDB id 1MKI, authors comment that observed phosphorylation by crystallography was not a consistent event and number of attempts to confirm this through mass spectrometry were apparently unsuccessful [34].

Out of the 15 conventional phosphorylation events within the eukaryotic proteins with rSASA value less than 0.3 in the PDB data set, kinase mediated autophosphorylation could account for 9 cases. Six other proteins were non-kinases in which three of the phosphosites record rSASA values between 0.2 and 0.25 (Additional file 1: Table S3) and three below 0.2. Based on this analysis a stringent criterion to determine the feasibility of a kinase mediated phosphorylation event would be rSASA of ≥0.3.With this stringent cut off, 2636 i.e., 44.3% phosphosites with rSASA values ≤0.3 will have to be considered as non-kinase mediated. Such a conclusion can be misleading because a) it overemphasizes the importance of structural information in analyzing experimentally determined phosphorylation sites; b) kinases recognize their phosphorylation sites from a dynamic population of protein conformations, and static crystal structures by and large do not account for such conformational freedom (exceptions are discussed below) and c) as mentioned before ~54% of experimentally determined phosphosites are in relatively less accessible regions of the protein (0.2-0.4). Based on the above arguments we decided that 0.2 would be the minimal rSASA requirement for a kinase mediated phosphorylation event.

With this limit we describe a conformational map under which all experimentally determined phosphorylation events can be grouped- a) disallowed region of phosphoconformation or zone of inaccessibility (with less than 0.2 rSASA) and b) allowed region of phosphoconformation or zone of accessibility (rSASA > 0.2). 75.4% or 4486 out of 5947 unique phosphorylation sites (with >70% amino acid coverage in the structure) from the PhosphoSitePlus belong to the allowed region of conformation and stand validated. 1461(24.6%) phosphosites belong to the disallowed region of conformational space. Compared to the phosphosite data, the number of matched structures available for the study is small (5947). Nevertheless the number of phosphorylation sites found within disallowed region of phosphoconformation (1461) is striking. If this observation were to be directly extrapolated to the total number of phosphorylation sites in the data base, ~53,770 phosphorylation events would fall under disallowed region of conformation (even with rSASA < 0.2, a low stringent cut off)! Substantial and vast conformational changes or even partial unfolding would be absolutely necessary to expose these phosphorylation sites to kinases! Therefore phosphorylation events within the disallowed region of phosphoconformation demand additional explanation/s.

In order to rule out the possibility that presence of these phosphorylation sites within inaccessible region may be a reflection of an inadvertent bias in the type and nature of the proteins that can be crystallized, phosphosites with and without PDB structures were classified under Panther pathway classification [35]. PDB seems to well represent most of the functional classes of proteins found within the phosphosite (Additional file 1: Table S4) indicating that our observations are not biased by the overabundance of a particular class of proteins. PhosphoSitePlus is a depository of both high and less well curated phosphorylation sites (CSA MS) from human and mouse [36]. To rule out the possibility that inaccessibility could be a reflection of level of curation we segregated this data into HTP MS with Pubmed reference only, CSA MS data only and those with records from both data set and classified them on the conformational map. Percent distribution of proteins within the disallowed region of conformation was not dependent on the level of curation (Additional file 1: Table S5) and for the rest of analysis all data are treated similarly.

### Conformational freedom in proteins

**Figure 3:**
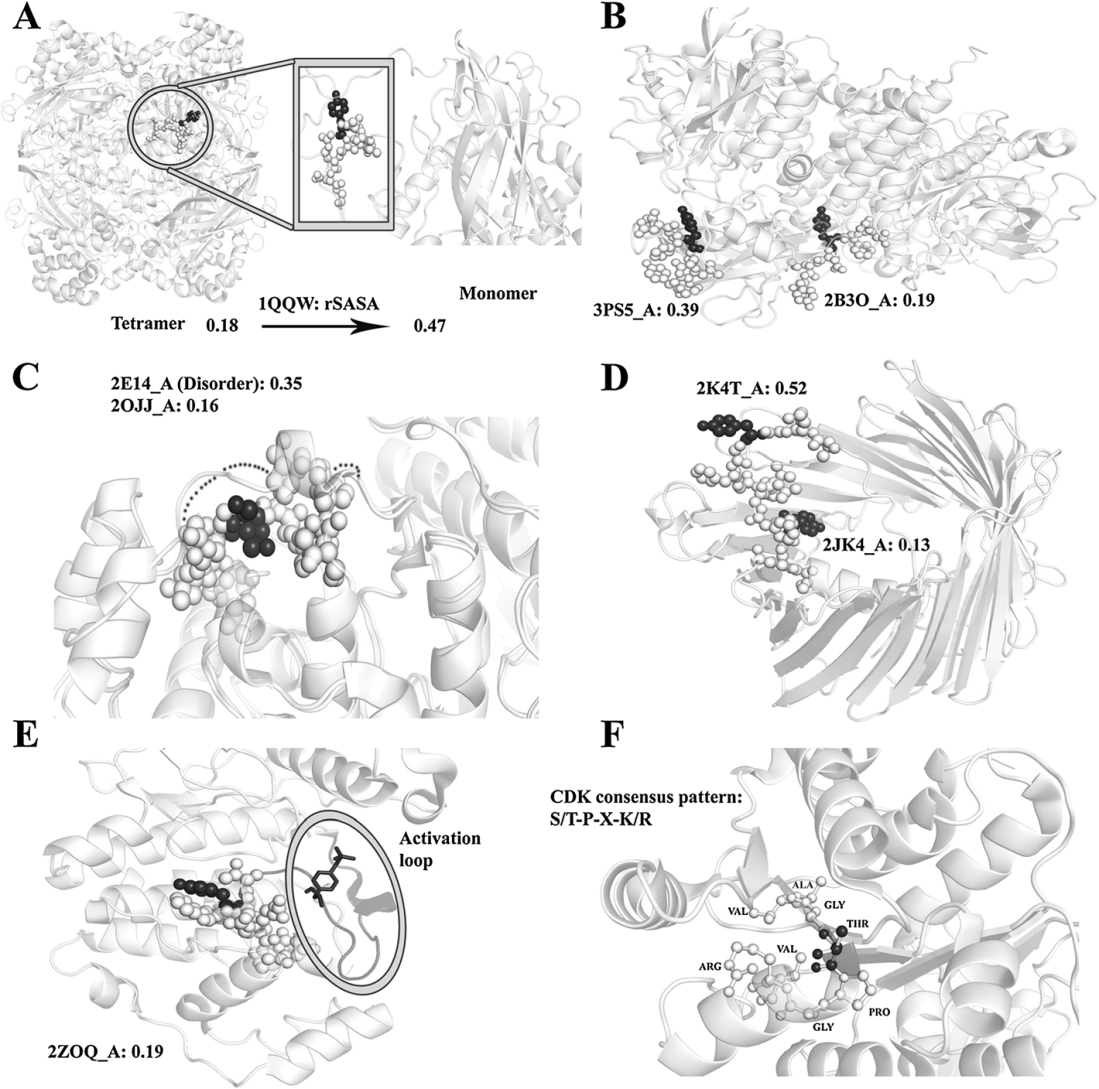
**Structures of a phosphosite from the disallowed and allowed region of conformation. A)** Phosphorylation site in Catalase, a homo tetramer (PDB ID: 1QQW) is buried at interface and has a calculated rSASA of 0.18. We extracted the monomeric form of this protein and recalculated the rSASA value which is now 0.47 indicating that the site is now accessible. **B)** Protein Tyrosine-protein phosphatase has two conformations in which the phosphorylation site is buried in an auto inhibited (PDB ID: 2B3O) conformation with a rSASA value of 0.19 and it is accessible to the solvent (rSASA of 0.39) in an open conformation (PDB ID: 3PS5). **C)** Two structures of Mitogen-activated protein kinase 1 are aligned where the phosphosite is present adjacent to a segment which is ordered in one PDB and is disordered (PDBID: 2E14) in another conformation. The disordered segment lacks coordinates for the overhanging loop. The phosphosite has greater rSASA (0.35) in the structure with the disordered segment than in the ordered structure (0.16). **D)** Two different conformations of Voltage-dependent anion-selective channel protein 1 in which the local structural changes were observed at the site of phosphorylation. In PDBID: 2JK4 phosphorylation site is buried inside channel (rSASA 0.13) whereas in PDBID: 2K4T phosphorylation the site is oriented outside (rSASA of 0.52). **E)** In Mitogen-activated protein kinase 3, the phosphosite is near to activation loop (rSASA 0.19). **F)** The phosphosite in Eukaryotic initiation factor has a known consensus pattern of S/T-P-X-K/R (rSASA 0.11) for Cyclin dependent kinase. The octapeptide is shown in ball and stick representation. In Figures **A**, **B**, **C**, **D**, **E** phosphosite is shown in ball and sphere notation and the phosphorylated residue is colored in black.

PDB is a redundant data base with crystal structures of the same protein solved multiple times under the same or different conditions [37,38]. As demonstrated by a number of studies these are store houses of information on dynamic changes in protein conformation [37,39]. Large scale changes in protein conformation have been well documented by solving structures of proteins in their different oligomeric forms or in their ligand bound conformation [40]. By the same arguments, phosphorylation sites within the disallowed region of conformation may be inaccessible because a) they are at the interface of a protein complex (Figure 3A). b) A sequence present in an inaccessible region of the protein (in the disallowed region of phosphoconformation) in one structure may be present in an accessible region of the protein (allowed region of phosphoconformation) in another structure (Figure 3B). For example in Figure 3B a phosphosite is buried to such an extent (PDB 2B3O) that in order to make this site accessible the protein will have to undergo large scale conformational change. PDB 3PS5 indeed shows that the sequence has moved to a different region of the protein surface where it is now accessible. In another example (PDB 2OJJ versus 2E14) order to disorder transition of the overhanging sequence captured in an alternate structure (Figure 3C), exposes residues to the solvent and therefore to a kinase. Small local structural changes can also expose a previously inaccessible site (Figure 3D). Conversely reverse changes in structure would make these sequences inaccessible. Such inherent dynamism in protein structure is likely to fine tune signal induced phosphorylation which requires many factors and several rounds of amplifications to reach the right target. c) Kinase induced autophosphorylation can happen in the inaccessible region of a protein where the active site is in proximity to the site of phosphorylation (Figure 3E). This exception however may not always be applicable as seen in the context FGFR kinase. Crystal structure of this protein revealed autophosphorylation at several tyrosine residues. One of them according to our definition is present in the disallowed region of conformation. Doubting whether such phosphorylation is relevant *in vivo* as this would require ‘unfolding’ of the protein [41] authors of the structure calculated the stoichiometry of phosphorylation at different sites. It turns out that the stoichiometry at the buried site was less than that seen in more permissible sites of the protein [42].

By removing structural constraints mentioned above and excluding kinases, the number of proteins in the disallowed region can be reduced from 24.6% to 13.5%. In addition if the sequence of a phosphosite matches with known sequence specificity of a kinase but the site were to be buried in a crystal structure one may assume that within the cellular milieu the site was probably accessible in an alternate conformation (Figure 3F). Exclusion of such sequences reduces the number of protein in the disallowed conformation to 10.8%.

The inherent dynamics of protein structures may also be sampled in an efficient manner using Cα-ENM Normal Mode Analysis as described in the Methods section. Since Cα-ENM Normal Mode Analysis is simple and computationally inexpensive, it provides a fast and automatable method to investigate the influence of protein conformation on rSASA and hence to further refine predictions about the accessibility of phosphorylation sites. This approach was tested on two groups of protein structures: a) four protein structures in which the proposed phosphorylation sites were classified as inaccessible on the basis of rSASA, but for which other structures had been reported with accessible phosphorylation sites; b) two proteins in which the phosphorylation sites were buried in all reported structures, which were selected as negative controls. For the two proteins in the negative control group (2AEB and 1GZ3), rSASA of all of the conformers sampled by Cα-ENM Normal Mode Analysis was less than 0.1, which supports the classification of these proteins in the disallowed region of phosphoconformation. By contrast, two of the four proteins from group A have low-energy conformers with rSASA >0.2 (2B3O and 3PY1). Since kinases recognize their phosphorylation sites from a dynamic population of protein conformations, our results suggests that these proteins can be reclassified in the allowed region of phosphoconformation. The remaining two proteins show significantly larger changes in rSASA with protein conformation than the negative control group, but there is not enough evidence to reclassify these proteins based on the computational results alone. The results of these simulations are summarized in the Supporting Information (Additional file 1: Table S6).

While the above analysis based on alternate structures and the normal mode analysis of proteins indicate that dynamism in protein structure can explain the apparent inaccessibility of a site, paucity of structural information of a large number of proteins, restricts direct extrapolation of this possibility to all phosphorylation events within a large data set like the PhosphoSitePlus. It is also unlikely that every MS derived data is a physiologically relevant identification. General knowledge on the physico chemical properties of proteins suggests that inaccessible and buried sequences are likely to be more hydrophobic than surface exposed sequences. This property was compared in phosphosites within allowed and disallowed region of conformation. Sequences within the disallowed regions were enriched in hydrophobic residues and correspondingly less in polar/charged residues (like aspartate or lysine), compared to those present in the allowed region of phosphoconformation (Additional file 1: Figure S3). If these sequences were to move and become exposed they are likely to render the protein unstable leading to aggregation. Additional experiments are therefore necessary to confirm phosphorylation at these sites. This is also reiterated by the normal mode analysis of two proteins with rSASA below 0.2 which showed that these sites are likely to remain inaccessible.

Since some of these phosphosites in the accessible region of the protein can be close to the protein surface which may allow ready access to the surface under certain conditions, we used an alternative program called DEPTH [43] which measures the distance between a given residue and the non bonded water molecules at the surface of the proteins. Farther the residue from the protein surface, larger is the DEPTH value. We found that while proteins with rSASA of >0.2 scored DEPTH values less than 30, those with rSASA less than 0.2 scored values greater than 30 indicating that they are indeed far removed from the surface (Additional file 1: Table S7).

**Figure 4:**
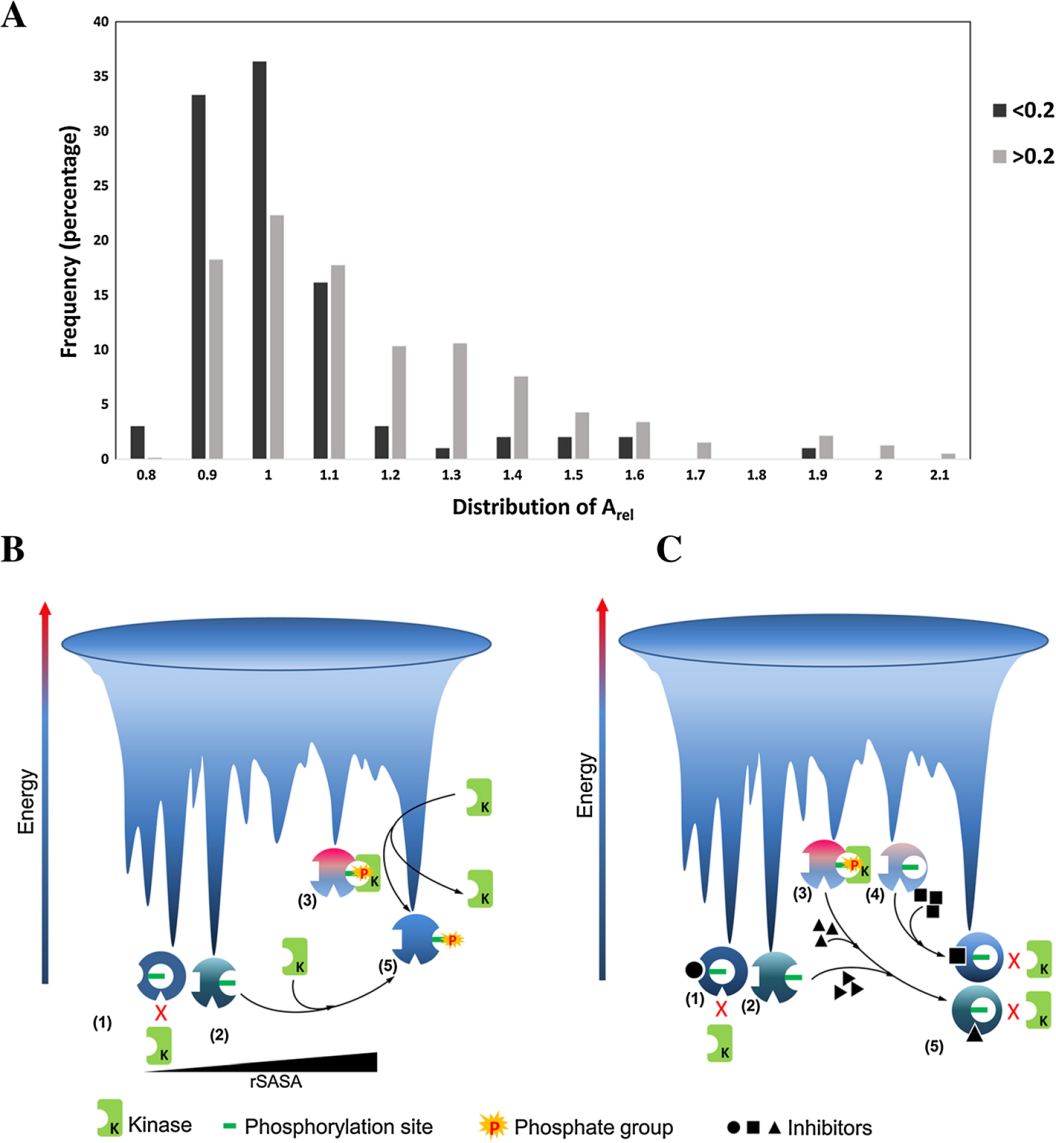
**Global conformational flexibility versus local accessibility of phosphosite and conformational trapping by inhibitors. A)** Protein flexibility or conformational changes were computed from the molecular weight of the proteins and compared with the experimentally determined SASA values for proteins from phosphosite data falling under disallowed (<0.2) and allowed region of phosphoconformation (>0.2). A_rel_ was calculated as the ratio of experimentally determined SASA to the predicted SASA. Dark bars indicate proteins in disallowed region of phosphoconformation while the light bars indicate those in the allowed region of conformation. **B)** The concept of conformational trapping by kinases vis a vis inhibitors is depicted using the free energy landscape. Protein in which the phosphosite is inaccessible is imagined to be in a low energy stable state (1) and the same site however may become accessible in a similar (2) or a high energy state (3). These conformations can be trapped by a kinase leading to phosphorylation (4) which stabilizes the protein and lowers its free energy. The phosphorylated ‘active’ state of the protein is shown here at a higher energy level (5) than the conformation in which the phosphosite is inaccessible. Action of a phosphatase may relieve the excess energy. **C)** This cartoon depicts conformational trapping by inhibitors. An inhibitor can bind to an allosteric site in the disallowed region of phosphoconformation freezing the protein in this kinase inaccessible state. Other inhibitors may bind to similar or higher energy conformations in which the phosphosite is increasingly accessible howoever inhibitor binding induces conformational changes rendering the phsophosite inaccessible to a kinase. The kinase accessible site may also be trapped by inhibitors competing at the phosphosite (not shown).

To determine if proteins containing phosphosites with small rSASA values (disallowed conformation) would differ from the others (allowed conformation) in terms of the extent to which they can undergo conformational changes, we used a predictive measure for conformational changes as reported by Marsh et al., [44]. Greater the deviation from the theoretical estimates larger is the conformational change. Results are shown in Figure 4A. A_rel_ greater than 1 reportedly indicates more flexibility or large conformational changes. Phosphosites within disallowed region show a higher frequency of distribution towards lesser A_rel_ as compared to those within the allowed region. Proteins in which the phosphosites are within the allowed region seem more flexible than those within the disallowed region of phosphoconformation. It is interesting to note that even though some proteins in which the phosphosites are in the disallowed region are conformationally more malleable (A_rel_ >0.1), the phosphosites themselves are inaccessible to a kinase indicating local conformational restriction.

While analyzing phosphorylation status of mitotic check point proteins, Durbin group observed that phosphorylation sites are less seen in structured regions of the proteins [29]. However they also observed that 15% of all phosphosites exhibited less than 10% solvent accessibility of their side chains in the unmodified form of the protein. They specifically describe such examples where these sites are found in buried regions of the protein and allude to the fact that these sites are likely to have problems in acting as substrates. More importantly they point out that local amino-acid repacking will be necessary to accommodate different electrostatic and steric properties between the unmodified and modified phosphorylation sites. They describe a few possibilities through which such sites can be exposed to kinase including intrinsic flexibility or an active conformational change induced by binding of other proteins, cofactors or ligand. These are in line with our observations.

It is in light of these observations that the following observations become very critical and intriguing. Many phosphorylation events within the phosphosite are seen at the interface of homo oligomeric proteins like a homo dimer or a tetramer [29,40]. If MS derived data is correct and physiologically relevant, then, phosphorylation must have occurred in the monomer which was accessible to the kinase under the experimental conditions. It is very difficult to prove the presence of monomeric forms of such proteins in cells. Due to their high affinity and sometimes interdependency of subunits to achieve the final folded form and stability, it is difficult to identify natively dissociated monomers in vitro. Such an attempt is often associated with protein unfolding or aggregation. On the other hand generation of such transient conformations in vivo may be coupled tightly with signaling events offering a great opportunity for the design of novel strategies to block kinase mediated signaling without targeting the kinase active site or their ATP binding site [45,46]. Data from alternate crystal structures indicate that it is possible to trap thermodynamically stable forms of conformations that are inaccessible to a kinase. One can imagine that an inhibitor may also bind to similar low energy conformation at an allosteric site, stabilizing the inaccessible/inactive conformation and prevent its transition into a kinase accessible form. These possibilities are represented in a cartoon form in Figure 4B and C. The cartoon is inspired by the popular and currently prevailing hypothesis that proteins exist as ensemble of conformers with varying degrees of free energy and one with the lowest energy is not necessarily the active form [47,48]. Presence of different conformers is envisaged to explain allostery, induced fit mechanisms and binding and selection by inhibitors some of which have been demonstrated by X-ray crystallography [49,50]. Many kinases more often than not are trapped in their inactive conformation by molecules that bind very close to the ATP binding site [51].

We imagine that the phosphosites within disallowed region of phosphoconformation sample conformational space of varying energy and the site may be accessible albeit in a transient high energy state or in a state of very similar energy (Figure 4B). A kinase may bind and trap these conformations and phosphorylate the protein. Inhibitors can bind to different states of the protein in this ensemble and freeze the protein in an inactive conformation (Figure 4C).

### Validation of an ambiguous data set

It would be useful if crystal structure information could help in better discrimination of false positive and false negative data. We used a very high quality data set [18] where scores were provided for strength or ambiguity in MS identification. An ‘A’ score of <19 indicated ambiguity but >19 indicated high confident assignment [18]. Many of these identifications had single phosphorylation while others carried up to three phosphorylations. We found matches for 72 of these phosphorylation events within the PDB. All but one site with a score of > 19 were in the allowed region of phosphorylation indicating a near perfect match between MS data and accessibility. In a single case with an A score of (>19) and PDB id 1I2M, the sequence was present in the disallowed region in all its available structures (Additional file 1: Table S8 and Figure S4A). Educated by our findings from the phosphosite data, we looked at this structure more carefully and found that the sequence was covered by an N terminus coil of 12 residues ranging from 24–35 numbering of PDB. If this region were dynamic, it would move away to readily expose these residues for phosphorylation. We deployed normal mode analysis and found that several of the low-energy Cα-ENM normal modes of 1I2M lead to conformers in which the N-terminal coil moves away from the phosphorylation site. For example, perturbation of the protein structure along the lowest-frequency normal mode results in a transition of the flap region from a closed to an open conformation. However, none of the protein conformations sampled by Cα-ENM normal mode analysis place this protein in the allowed region of phosphoconformation (Additional file 1: Table S6). In the absence of support from existing structures it would be worthwhile to do additional experiments to monitor phosphorylation at this site using other independent techniques. It is noteworthy that this protein is cylindrical in shape with the octapeptide that surrounds the phosphorylation site lying length ways along the inside of the cylinder, which makes it relatively inaccessible to kinases. If the identification is indeed correct then it is very likely that a conformational change has been introduced in the protein under the experimental conditions by signaling or via protein-protein or protein-ligand interactions to name a few. Or the kinase may itself induce the necessary conformational change in the protein upon binding.

Out of eight structures with an A score of <19, and considered ambiguous, seven belong to allowed region of phosphorylation (Additional file 1: Table S6 and Figure S4B). Our analysis indicates that these phosphorylation events are structurally feasible and are probably a physiological reality. We were fortunate to use these proteins because the authors themselves placed these proteins under the ambiguous category. As mentioned before there are several other phosphosites in Phsophosite plus that belong to the disallowed region of conformation that needs to be verified by other additional experiments.

## Conclusions

In summary a very high percentage of MS data obeys common and inherent laws of protein structure required for kinase access. In a significant percentage of cases this rule does not holds true. For a few cases, in depth structural analysis and knowledge about conformational freedom and presence of redundant protein structures allows us to explain the discrepancy. Our detailed analysis of the PDB structures of phosphorylated sequences caution that sites within inaccessible regions of a protein are mostly biologically irrelevant or non-kinase mediated (Additional file 1: Table S3), enriched in hydrophobic residues prone to aggregation upon exposure. It would definitely be worthwhile to revisit the mass spectrometric data in such conflicting cases and further experiments may be needed to decide whether these are actually a kinase driven reaction or not. It is also important to use complementary techniques like NMR, sophisticated MD simulations other biophysical studies to better understand the fate of buried residues in post translational modifications [27,52].

A phosphorylation event within disallowed region of conformation is a true physiologically relevant conformation trapped by a kinase if a) an alternate structure is present where the site is accessible, and b) the sites have been identified by many independent investigators under different experimental conditions c) accuracy of detection has been ensured. It is possible to think of inhibitors which like the kinase, may trap such altered conformations that will prevent either a kinase mediated phosphorylation or inhibit protein-protein interaction leading to phenotypic consequences. These may be exploited for the design of inhibitors to prevent overactive kinase mediated signaling in diseases such as cancer. Such a strategy will be a viable alternative to currently available methods that target kinases which are however prone to mutational changes and therefore drug resistance leading to relapses or more severe forms of the disease.

## Methods

### PhosphoSitePlus dataset

A total of 2,18,870 phosphosites were downloaded from Phosphosite-Plus database. From this source data set, 32,609 unique accession numbers covering all databases like Uniprot, NCBI, Ensembl were shortlisted. Independent match with the well curated Uniprot/SwissProt database fetched 27,678 unique accession numbers. The remaining 4931 entries failed because they were either isoforms or proteins are listed under Uniprot/Trembl, NCBI, and Ensembl databases.

#### Matching phosphorylation sites from PhosphoSitePlus against the PDB

From these 27,678 shortlisted proteins, those with available PDB structures were searched using a Perl script. Entries were available for 5131 proteins corresponding to 54,348 phosphosites. In phosphositeplus these sites are represented in a 15 residue format such as XXXXXXX(S/T/Y)XXXXXXX. These were extracted and trimmed further to obtain octapeptides of the form XXX(S/T/Y)XXXX which were further analysed. Co-ordinates for these octapeptides could be located for 16,528 phosphorylation sites in 3,758 proteins. For the other 37,820 sites, the octapeptide sequences were not covered in the solved PDB structure and were not considered further.

All the PDB files for the matched proteins were downloaded using download files tool available on PDB website (http://www.rcsb.org/pdb/download/download.do). PDB IDs were provided as input. Out of 16,528 phosphorylation sites, matches for 4162 sites failed because co-ordinates for some or all of the eight residues (disordered) were either absent or there was a mutation within the octapeptide sequence. In order to distinguish between the two possibilities, mutated residues were searched by a modified query. Here residue at each position was replaced by 19 other amino acids one at a time. These modified sequences were then matched back to the parent sequence. For example for the octapeptide ATGSELVD, the query sequence was altered by Mut1: XTGSELVD, Mut2: AXGSELVD, Mut3: ATXSELVD, Mut4: ATGXELVD, Mut5: ATGSXLVD, Mut6: ATGSEXVD, Mut7: ATGSELXD or Mut8: ATGSELVX where X is any one of the other 19 amino acids. By this method we could clearly differentiate between mutation and disordered regions. 583 sites carried a mutant sequence and 3579 sites were disordered.

### Relative solvent accessible surface area of octapeptide (rSASA)

To ensure reliability, we limited our analysis to proteins for which structures were determined for at least 70% of the primary sequence. 5947 phosphorylation sites met with this criterion and were analyzed further using a stand-alone software called POPS for Parameter Optimised Surfaces [53]. rSASA for each octapeptide from PhosphoSitePlus were extracted from the corresponding complete files using perl scripts. The rSASA for each octapeptide was calculated as an average of %SASA which is the ratio between solvent accessible surface area (SASA) of a residue in its three dimensional structure and SASA of its extended tripeptide (Gly/Ala-X-Gly/Ala) conformation. To see whether energy minimization of PDB structures has any effect on calculated rSASA value, a set of 201 high resolution (> = 3.0) structures (covering 568 phosphosites) were energy minimized using GROMACS software with OPLS force field [54]. Residue level rSASA values were also calculated for S/T/Y phosphorylation for both PDB and Phosphosite data.

To obtain a measure of conformational flexibility of proteins containing phosphosites with <0.2 rSASA and those with >0.2 rSASA, a predictive measure of relative solvent accessibility (A_rel_) as reported by from Marsh et al. [44], was used. Only monomers from our phosphosite dataset were considered and those with greater than 5 disordered residues were filtered. Observed SASA of a structure is calculated from AREAIMOL application from CCP4 package and predicted SASA is calculated by using Equation 4.44*M^0.77 where M is the mass of the protein. A_rel_ = observed SASA/predicted SASA calculated as above.

### Secondary structure determination

Similar protocol was followed for secondary structure determination using Stride stand-alone tool [55]. A representative secondary structure of the octapeptide would be HHTTEEC (helix-helix-turn-turn-sheet-sheet-coil).

### Preparation of PDB dataset containing phosphorylated residues

PDB database was searched for a ‘**modified residue**’ within advanced search interface page provided at http://www.rcsb.org/pdb/search/advSearch.do. This search produced an output of 16436 structures. Using perl scripts and searching specifically for Serine, Threonine, Tyrosine, Histidine, Cysteine, Aspartic acid residues a total of 1230 phosphorylated structures were fetched.

If multiple chains were phosphorylated in the homo multimeric structures then a single chain was considered. Redundant structures were removed and only unique phosphosites were considered which resulted in 280 proteins. Out of this 280, 104 are phosphoserine, 76 are phosphothreonine, 77 are phosphotyrosine, 19 are phosphohistidine, 3 are phosphocysteine residues and one of them is aspartyl phosphate.

### Cα elastic network model - normal mode analysis

To investigate the influence of protein conformation on rSASA, we used normal mode analysis of a Cα-Elastic Network Model (Cα-ENM) of protein structure to sample thermally accessible low-energy conformational states. A Cα-ENM is a coarse-grained model of protein structure in which each residue is represented by a single point located at its Cα atom coordinate [56,57]. Pairwise interactions between these coarse-grained points are computed using a harmonic potential of the form:

$$E=\frac{1}{2}k\sum_{d_{\mathrm{ij}}^{0}<R_{c}}^{N}\left(d_{\mathrm{ij}}-d_{\mathrm{ij}}^{0}\right)$$

where k is the force-constant, d^0^_ij_ is the distance between Cα atoms i and j in the crystal structure of the protein, d_ij_ is the distance between these points in the elastic network model, R_C_ is a distance cut-off such that only Cα atoms that are separated by less than this distance contribute to the potential energy, and N is the number of Cα atoms in the protein. In accordance with previous studies [58], we use R = 10 Å and a single value of k for all residues. The input coordinates of the Cα atoms of each protein were obtained from the RCSB PDB [59] with missing residues added using the program Prime. Once a Cα - ΕΝΜ has been defined for a protein, harmonic vibrational analysis can be performed using standard tools [60] leading to 3 N-6 eigenvectors (“normal modes”) with non-zero eigenvalues. Although Cα - ΕΝΜs are conceptually simple, there is substantial evidence that their low-frequency (low-eigenvalue) normal modes are useful for studying large-scale conformational changes in proteins, and they have been used successfully in a wide-variety of applications, including biasing molecular dynamics simulations [61], sampling protein-flexibility in molecular docking [62], mapping conformational transitions [58], and fitting of atomic structures into low-resolution electron density maps [63].

Once the normal modes had been calculated from the Cα Elastic Network Model, a set of discrete molecular conformations were generated for each protein by permuting the crystal structure at small intervals along each of the twenty lowest-energy normal modes. The value of rSASA was then calculated for each of the discrete molecular conformations using the POPS software as discussed previously. In total, 401 distinct low-energy molecular conformers were generated for each protein (20 normal modes * 20 conformers per mode + 1 input structure = 401 conformers). Since Cα-ENM Normal Mode Analysis is simple and computationally inexpensive, it provides a fast and automatable method to investigate the influence of protein conformation on rSASA and hence to further refine predictions about the accessibility of phosphorylation sites.

## Abbreviations

PDB: Protein data bank; POPS: Parameter optimised surfaces; SASA: Solvent accessible surface area; rSASA: Relative solvent accessible surface area; ENM: Elastic network model.

## Competing interests

The authors declare that they have no competing interests.

## Supplementary Material

Additional file 1: Figure S1 Schema/work flow of analysis of Phosphosites. **Figure S2**. Accessibility of Phosphosites before and after energy minimization of the corresponding protein structures. **Figure S3**. Physico chemical properties of amino acids within the posphosites in allowed and disallowed region of conformation. **Figure S4**. Structure of Phosphosites within the ambiguous dataset. **Table S1**. Accessibility of Phosphosites from the PhosphoSitePlus Data base. **Table S2.** Accessibility of octapeptides carrying the phosphorylated residue in eukaryotic and prokaryotic proteins within the PDB database. **Table S3**. Nature and origin of Phosphosites (S/T/Y) with rSASA values less than 0.3 within the PDB data set of eukaryotic proteins. **Table S4**. Functional Classification of Phosphosites. **Table S5**. Extent of accessibility of phosphosites with different levels of curation. **Table S6**. Statistics for the Cα-Elastic Network Model Normal Mode Analyses of selected proteins. **Table S7**. DEPTH values an alternate measure of accessibility of phosphosites. **Table S8**. Accessibility as a measure of confidence in the identification of phosphosites. Click here for file

## References

[B1] Graves JD Krebs EG Protein phosphorylation and signal transduction Pharmacol Ther 1999 82 2–3 111 121 10454190 10.1016/s0163-7258(98)00056-4

[B2] Malumbres M Barbacid M Cell cycle kinases in cancer Curr Opin Genet Dev 2007 17 1 60 65 10.1016/j.gde.2006.12.008 17208431

[B3] Harashima H Dissmeyer N Schnittger A Cell cycle control across the eukaryotic kingdom Trends Cell Biol 2013 7 345 356 23566594 10.1016/j.tcb.2013.03.002

[B4] Johnson LN Barford D The effects of phosphorylation on the structure and function of proteins Annu Rev Biophys Biomol Struct 1993 22 199 232 10.1146/annurev.bb.22.060193.001215 8347989

[B5] Olsen JV Blagoev B Gnad F Macek B Kumar C Mortensen P Mann M Global, in vivo, and site-specific phosphorylation dynamics in signaling networks Cell 2006 127 3 635 648 10.1016/j.cell.2006.09.026 17081983

[B6] Dengjel J Akimov V Olsen JV Bunkenborg J Mann M Blagoev B Andersen JS Quantitative proteomic assessment of very early cellular signaling events Nat Biotechnol 2007 25 5 566 568 10.1038/nbt1301 17450129

[B7] Kreegipuu A Blom N Brunak S PhosphoBase, a database of phosphorylation sites: release 2.0 Nucleic Acids Res 1999 27 1 237 239 10.1093/nar/27.1.237 9847189 PMC148144

[B8] Hubbard MJ Cohen P On target with a new mechanism for the regulation of protein phosphorylation Trends Biochem Sci 1993 18 5 172 177 10.1016/0968-0004(93)90109-Z 8392229

[B9] Bodenmiller B Aebersold R Phosphoproteome resource for systems biology research Methods Mol Biol 2011 694 307 322 10.1007/978-1-60761-977-2_19 21082442

[B10] Aebersold R Mann M Mass spectrometry-based proteomics Nature 2003 422 6928 198 207 10.1038/nature01511 12634793

[B11] Carretero J Shimamura T Rikova K Jackson AL Wilkerson MD Borgman CL Buttarazzi MS Sanofsky BA McNamara KL Brandstetter KA Walton ZE Gu TL Silva JC Crosby K Shapiro GI Maira SM Ji H Castrillon DH Kim CF García-Echeverría C Bardeesy N Sharpless NE Hayes ND Kim WY Engelman JA Wong KK Integrative genomic and proteomic analyses identify targets for Lkb1-deficient metastatic lung tumors Cancer Cell 2010 17 6 547 559 10.1016/j.ccr.2010.04.026 20541700 PMC2901842

[B12] Carr SA Annan RS Huddleston MJ Mapping posttranslational modifications of proteins by MS-based selective detection: application to phosphoproteomics Methods Enzymol 2005 405 82 115 16413312 10.1016/S0076-6879(05)05005-6

[B13] Annan RS Carr SA The essential role of mass spectrometry in characterizing protein structure: mapping posttranslational modifications J Protein Chem 1997 16 5 391 402 10.1023/A:1026384605285 9246619

[B14] Mann M Jensen ON Proteomic analysis of post-translational modifications Nat Biotechnol 2003 21 3 255 261 10.1038/nbt0303-255 12610572

[B15] Kim JE Tannenbaum SR White FM Global phosphoproteome of HT-29 human colon adenocarcinoma cells J Proteome Res 2005 4 4 1339 1346 10.1021/pr050048h 16083285

[B16] Perkins DN Pappin DJ Creasy DM Cottrell JS Probability-based protein identification by searching sequence databases using mass spectrometry data Electrophoresis 1999 20 18 3551 3567 10.1002/(SICI)1522-2683(19991201)20:18<3551::AID-ELPS3551>3.0.CO;2-2 10612281

[B17] MacCoss MJ Wu CC Yates JR 3rd Probability-based validation of protein identifications using a modified SEQUEST algorithm Anal Chem 2002 74 21 5593 5599 10.1021/ac025826t 12433093

[B18] Beausoleil SA Villen J Gerber SA Rush J Gygi SP A probability-based approach for high-throughput protein phosphorylation analysis and site localization Nat Biotechnol 2006 24 10 1285 1292 10.1038/nbt1240 16964243

[B19] Gevaert K Staes A Van Damme J De Groot S Hugelier K Demol H Martens L Goethals M Vandekerckhove J Global phosphoproteome analysis on human HepG2 hepatocytes using reversed-phase diagonal LC Proteomics 2005 5 14 3589 3599 10.1002/pmic.200401217 16097034

[B20] Rush J Moritz A Lee KA Guo A Goss VL Spek EJ Zhang H Zha XM Polakiewicz RD Comb MJ Immunoaffinity profiling of tyrosine phosphorylation in cancer cells Nat Biotechnol 2005 23 1 94 101 10.1038/nbt1046 15592455

[B21] Ballif BA Villen J Beausoleil SA Schwartz D Gygi SP Phosphoproteomic analysis of the developing mouse brain Mol Cell Proteomics 2004 3 11 1093 1101 10.1074/mcp.M400085-MCP200 15345747

[B22] Olsen JV Vermeulen M Santamaria A Kumar C Miller ML Jensen LJ Gnad F Cox J Jensen TS Nigg EA Brunak S Mann M Quantitative phosphoproteomics reveals widespread full phosphorylation site occupancy during mitosis Sci Signal 2010 3 104 ra3 20068231 10.1126/scisignal.2000475

[B23] Mann M Ong SE Gronborg M Steen H Jensen ON Pandey A Analysis of protein phosphorylation using mass spectrometry: deciphering the phosphoproteome Trends Biotechnol 2002 20 6 261 268 10.1016/S0167-7799(02)01944-3 12007495

[B24] Ficarro SB McCleland ML Stukenberg PT Burke DJ Ross MM Shabanowitz J Hunt DF White FM Phosphoproteome analysis by mass spectrometry and its application to Saccharomyces cerevisiae Nat Biotechnol 2002 20 3 301 305 10.1038/nbt0302-301 11875433

[B25] Groban ES Narayanan A Jacobson MP Conformational changes in protein loops and helices induced by post-translational phosphorylation PLoS Comput Biol 2006 2 4 e32 10.1371/journal.pcbi.0020032 16628247 PMC1440919

[B26] Kitchen J Saunders RE Warwicker J Charge environments around phosphorylation sites in proteins BMC Struct Biol 2008 8 19 10.1186/1472-6807-8-19 18366741 PMC2291461

[B27] Latzer J Shen T Wolynes PG Conformational switching upon phosphorylation: a predictive framework based on energy landscape principles Biochemistry 2008 47 7 2110 2122 10.1021/bi701350v 18198897

[B28] Iakoucheva LM Radivojac P Brown CJ O'Connor TR Sikes JG Obradovic Z Dunker AK The importance of intrinsic disorder for protein phosphorylation Nucleic Acids Res 2004 32 3 1037 1049 10.1093/nar/gkh253 14960716 PMC373391

[B29] Jimenez JL Hegemann B Hutchins JR Peters JM Durbin R A systematic comparative and structural analysis of protein phosphorylation sites based on the mtcPTM database Genome Biol 2007 8 5 R90 10.1186/gb-2007-8-5-r90 17521420 PMC1929158

[B30] Russo AA Jeffrey PD Pavletich NP Structural basis of cyclin-dependent kinase activation by phosphorylation Nat Struct Biol 1996 3 8 696 700 10.1038/nsb0896-696 8756328

[B31] Espinoza-Fonseca LM Kast D Thomas DD Thermodynamic and structural basis of phosphorylation-induced disorder-to-order transition in the regulatory light chain of smooth muscle myosin J Am Chem Soc 2008 130 37 12208 12209 10.1021/ja803143g 18715003 PMC2875193

[B32] Venkatraman P Balakrishnan S Rao S Hooda Y Pol S A sequence and structure based method to predict putative substrates, functions and regulatory networks of endo proteases PLoS One 2009 4 5 e5700 10.1371/journal.pone.0005700 19492082 PMC2683571

[B33] Barkan DT Hostetter DR Mahrus S Pieper U Wells JA Craik CS Sali A Prediction of protease substrates using sequence and structure features Bioinformatics 2010 26 14 1714 1722 10.1093/bioinformatics/btq267 20505003 PMC2894511

[B34] Brown G Singer A Proudfoot M Skarina T Kim Y Chang C Dementieva I Kuznetsova E Gonzalez CF Joachimiak A Savchenko A Yakunin AF Functional and structural characterization of four glutaminases from Escherichia coli and Bacillus subtilis Biochemistry 2008 47 21 5724 5735 10.1021/bi800097h 18459799 PMC2735108

[B35] Mi H Muruganujan A Casagrande JT Thomas PD Large-scale gene function analysis with the PANTHER classification system Nat Protoc 2013 8 8 1551 1566 10.1038/nprot.2013.092 23868073 PMC6519453

[B36] Hornbeck PV Kornhauser JM Tkachev S Zhang B Skrzypek E Murray B Latham V Sullivan M PhosphoSitePlus: a comprehensive resource for investigating the structure and function of experimentally determined post-translational modifications in man and mouse Nucleic Acids Res 2012 40 Database issue D261 D270 22135298 10.1093/nar/gkr1122 PMC3245126

[B37] Zhang Y Stec B Godzik A Between order and disorder in protein structures: analysis of "dual personality" fragments in proteins Structure 2007 15 9 1141 1147 10.1016/j.str.2007.07.012 17850753 PMC2084070

[B38] Wang W Liu L Song X Mo Y Komma C Bellamy HD Zhao ZJ Zhou GW Crystal structure of human protein tyrosine phosphatase SHP-1 in the open conformation J Cell Biochem 2011 112 8 2062 2071 10.1002/jcb.23125 21465528 PMC3135737

[B39] Low C Homeyer N Weininger U Sticht H Balbach J Conformational switch upon phosphorylation: human CDK inhibitor p19INK4d between the native and partially folded state ACS Chem Biol 2009 4 1 53 63 10.1021/cb800219m 19063602

[B40] Gunasekaran K Nussinov R How different are structurally flexible and rigid binding sites? Sequence and structural features discriminating proteins that do and do not undergo conformational change upon ligand binding J Mol Biol 2007 365 1 257 273 10.1016/j.jmb.2006.09.062 17059826

[B41] Mohammadi M Schlessinger J Hubbard SR Structure of the FGF receptor tyrosine kinase domain reveals a novel autoinhibitory mechanism Cell 1996 86 4 577 587 10.1016/S0092-8674(00)80131-2 8752212

[B42] Furdui CM Lew ED Schlessinger J Anderson KS Autophosphorylation of FGFR1 kinase is mediated by a sequential and precisely ordered reaction Mol Cell 2006 21 5 711 717 10.1016/j.molcel.2006.01.022 16507368

[B43] Chakravarty S Varadarajan R Residue depth: a novel parameter for the analysis of protein structure and stability Structure 1999 7 7 723 732 10.1016/S0969-2126(99)80097-5 10425675

[B44] Marsh JA Buried and accessible surface area control intrinsic protein flexibility J Mol Biol 2013 425 17 3250 3263 10.1016/j.jmb.2013.06.019 23811058

[B45] Zhang J Yang PL Gray NS Targeting cancer with small molecule kinase inhibitors Nat Rev Cancer 2009 9 1 28 39 10.1038/nrc2559 19104514 PMC12406740

[B46] Chan WW Wise SC Kaufman MD Ahn YM Ensinger CL Haack T Hood MM Jones J Lord JW Lu WP Miller D Patt WC Smith BD Petillo PA Rutkoski TJ Telikepalli H Vogeti L Yao T Chun L Clark R Evangelista P Gavrilescu LC Lazarides K Zaleskas VM Stewart LJ Van Etten RA Flynn DL Conformational control inhibition of the BCR-ABL1 tyrosine kinase, including the gatekeeper T315I mutant, by the switch-control inhibitor DCC-2036 Cancer Cell 2011 19 4 556 568 10.1016/j.ccr.2011.03.003 21481795 PMC3077923

[B47] Lee GM Craik CS Trapping moving targets with small molecules Science 2009 324 5924 213 215 10.1126/science.1169378 19359579 PMC2981433

[B48] Teague SJ Implications of protein flexibility for drug discovery Nat Rev Drug Discovery 2003 2 7 527 541 10.1038/nrd1129 12838268

[B49] Kar G Keskin O Gursoy A Nussinov R Allostery and population shift in drug discovery Curr Opin Pharmacol 2010 10 6 715 722 10.1016/j.coph.2010.09.002 20884293 PMC7316380

[B50] Hardy JA Wells JA Searching for new allosteric sites in enzymes Curr Opin Pharmacol 2004 14 6 706 715 10.1016/j.sbi.2004.10.009 15582395

[B51] Azam M Latek RR Daley GQ Mechanisms of autoinhibition and STI-571/imatinib resistance revealed by mutagenesis of BCR-ABL Cell 2003 112 6 831 843 10.1016/S0092-8674(03)00190-9 12654249

[B52] Volkman BF Lipson D Wemmer DE Kern D Two-state allosteric behavior in a single-domain signaling protein Science 2001 291 5512 2429 2433 10.1126/science.291.5512.2429 11264542

[B53] Cavallo L Kleinjung J Fraternali F POPS: a fast algorithm for solvent accessible surface areas at atomic and residue level Nucleic Acids Res 2003 31 13 3364 3366 10.1093/nar/gkg601 12824328 PMC169007

[B54] Pronk S Pall S Schulz R Larsson P Bjelkmar P Apostolov R Shirts MR Smith JC Kasson PM van der Spoel D Hess B Lindahl E GROMACS 4.5: a high-throughput and highly parallel open source molecular simulation toolkit Bioinformatics 2013 29 7 845 854 10.1093/bioinformatics/btt055 23407358 PMC3605599

[B55] Heinig M Frishman D STRIDE: a web server for secondary structure assignment from known atomic coordinates of proteins Nucleic Acids Res 2004 32 Web Server issue W500-502 15215436 10.1093/nar/gkh429 PMC441567

[B56] Tirion MM Large amplitude elastic motions in proteins from a single-parameter, atomic analysis Phys Rev Lett 1996 77 9 1905 1908 10.1103/PhysRevLett.77.1905 10063201

[B57] Hinsen K Analysis of domain motions by approximate normal mode calculations Proteins 1998 33 3 417 429 10.1002/(SICI)1097-0134(19981115)33:3<417::AID-PROT10>3.0.CO;2-8 9829700

[B58] Zheng W Brooks BR Hummer G Protein conformational transitions explored by mixed elastic network models Proteins 2007 69 1 43 57 10.1002/prot.21465 17596847

[B59] Bernstein FC Koetzle TF Williams GJ Meyer EF Jr Brice MD Rodgers JR Kennard O Shimanouchi T Tasumi M The protein data bank. A computer-based archival file for macromolecular structures Eur J Biochem 1977 80 2 319 324 10.1111/j.1432-1033.1977.tb11885.x 923582

[B60] Palmer DS Jensen F Predicting large-scale conformational changes in proteins using energy-weighted normal modes Proteins 2011 79 10 2778 2793 10.1002/prot.23105 21905106

[B61] Isin B Schulten K Tajkhorshid E Bahar I Mechanism of signal propagation upon retinal isomerization: insights from molecular dynamics simulations of rhodopsin restrained by normal modes Biophys J 2008 95 2 789 803 10.1529/biophysj.107.120691 18390613 PMC2440475

[B62] Akten ED Cansu S Doruker P A docking study using atomistic conformers generated via elastic network model for cyclosporin A/cyclophilin A complex J Biomol Struct Dyn 2009 27 1 13 26 10.1080/07391102.2009.10507292 19492859

[B63] Tama F Miyashita O Brooks CL 3rd Flexible multi-scale fitting of atomic structures into low-resolution electron density maps with elastic network normal mode analysis J Mol Biol 2004 337 4 985 999 10.1016/j.jmb.2004.01.048 15033365

