# Supplementary material for: Structural interrogation of phosphoproteome identified by mass spectrometry reveals allowed and disallowed regions of phosphoconformation
Source: BMC Struct Biol. 2014 Mar 11;14:9. doi: 10.1186/1472-6807-14-9 (PMC4007652; doi:10.1186/1472-6807-14-9)
Supplement: Additional file 1: Figure S1 — Schema/work flow of analysis of Phosphosites. Figure S2. Accessibility of Phosphosites before and after energy minimization of the corresponding protein structures. Figure S3. Physico chemical properties of amino acids within the posphosites in allowed and disallowed region of conformation. Figure S4. Structure of Phosphosites within the ambiguous dataset. Table S1. Accessibility of Phosphosites from the PhosphoSitePlus Data base. Table S2. Accessibility of octapeptides carrying the phosphorylated residue in eukaryotic and prokaryotic proteins within the PDB database. Table S3. Nature and origin of Phosphosites (S/T/Y) with rSASA values less than 0.3 within the PDB data set of eukaryotic proteins. Table S4. Functional Classification of Phosphosites. Table S5. Extent of accessibility of phosphosites with different levels of curation. Table S6. Statistics for the Cα-Elastic Network Model Normal Mode Analyses of selected proteins. Table S7. DEPTH values an alternate measure of accessibility of phosphosites. Table S8. Accessibility as a measure of confidence in the identification of phosphosites. [file 1472-6807-14-9-S1.zip › 1695290998113254_TableS3.doc]

**Supplementary Table 3:** Nature and origin of Phosphosites (S/T/Y) with rSASA values less than 0.3 within the PDB data set of eukaryotic proteins.

| **Uniprot ID** | **PDB ID** | **Position of Phosphorylated Residue** | **Octapepide Sequence** | **rSASA** | **Secondary Structure** | **Comments** |
| --- | --- | --- | --- | --- | --- | --- |
| P52956 | 3QPD | 126 | GGYSQGTA | 0.09 | EECCHHHH | Cutinase crystalized with paraoxon, Not in pubmed |
| Q9KWY4 | 2W5X | 84 | VTDSASGA | 0.112857 | TTCCHHHH | Alkaline phosphatase, dimer, S84 in catalytic pocket, phosphate presumably originates from PEG |
| P00634 | 2GA3 | 102 | VTDTAASA | 0.124286 | TTCCHHHH | Alkaline phosphatase active site mutant S102T |
| P05187 | 2GLQ | 92 | VPDSGATA | 0.131429 | TTCCHHHH | Placental alkaline phosphatase (PLAP) crystalized with Strontium |
| P00634 | 1HJK | 102 | VTDSAASA | 0.132857 | TTCCHHHH | Mutant alkaline phosphatase, phospho-enzyme intermediate was trapped |
| 13363950 | 2FEW | 384 | IVASDAGM | 0.151429 | EEEETTTC | Active site of Mannitol transporter (phosphotransferase) C384 was mutated to S to capture phosphorylated state. Two active site domains are connected through flexible linker |
| P00949 | 1JDY | 116 | LTASHNPG | 0.171429 | ECTTTTCC | Phoshoglucomutase (phosphotrasferase)  Not recorded in Pubmed |
| Q9F4L3 | 3IAF | 28 | LHGSHIDT | 0.172857 | CCTTTHHH | A28S mutant of thiamin diphosphate dependent Benzaldehyde lyase |
| O28025 | 3AMU | 18 | GMCTTYLA | 0.174286 | TCCCHHHH | tRNA (Ile2) 2-agmatinylcytidine synthetase TiaS, Auto phosphorylated at T18 and it is close to ATP triphosphate |
| P25924 | 1PJQ | 128 | AVSSGGTS | 0.178571 | EEEECTTT | Siroheme synthase, dimer, S128 phosphorylation inhibits its dehydrogenase activity |
| Q818Z9 | 3UO0 | 85 | GKDTMTGH | 0.19 | CCCCHHHH | Phosphopentomutase, Phosphorylated active site T85 is a trapped intermediate |
| B0V9T6 | 2XKK | 124 | AMRYTEAK | 0.191429 | TTTTTEEE | Fusion of different subunit of Topoisomerase IV with DNA |
| D0B6G5 | 4HJH | 104 | VTGSHIPD | 0.2 | ECTTTTTT | Phosphoglucomutase.  Not in Pubmed |
| P08877 | 3OQO | 46 | NLKSIMGV | 0.21 | TEEEECCH | Phosphocarrier protein HPr in complex with DNA and other protein. Not in PubMed |
| O69250 | 2NZV | 46 | NLKSIMGV | 0.214286 | ECCCCCHH | Phosphocarrier protein HPr. Reported in several papers |
| C9QXS7 | 3PX7 | 319 | YITYMRTD | 0.215714 | CBCCCCCC | DNA topoisomerase. Not in PubMed. |
| P05132 | 3FHI | 197 | RTWTLAGT | 0.22 | TBCCCEEC | PKA c-alpha, T197 and T246 in catalytic loop compete with cAMP for phosphate binding cassette in RI alpha |
| A1L1M0 | 3TNQ | 197 | RTWTLCGT | 0.227143 | TBCCCEEC | cAMP-dependent protein kinase catalytic subunit alpha, T97 and T139 are in catalytic subunit. It is a tetramer (Dimer of dimer ) |
| P35557 | 3QIC | 411 | VDGSVYKL | 0.23 | EETTTTTT | E339K mutant Glucokinase may be autophosphorylated due to which ATP binding was blocked. Supported by MS |
| P26276 | 1PCM | 108 | LTGSHNPP | 0.232857 | ECCCCCTT | Phosphoglucomutase crystals were grown in Para nitrophenyl phosphate. Not discussed in the manuscript |
| Q5NII8 | 3I3W | 101 | ITASHNKF | 0.232857 | ECTTTTTT | Phosphoglucosamine Mutase. Not in PubMed. |
| O06976 | 2AK7 | 46 | NAKSIMGL | 0.237143 | ECCCHHHH | Domain-swapped dimer of Ser46-phosphorylated HPr-like protein Crh |
| P11387 | 1RRJ | 723 | KLSYLDPR | 0.24 | HHHHCCHH | Camptothecin-resistant (F361S and N722S) DNA topoisomerase I in complex with DNA and topotecan |
| Q79PF4 | 3S1A | 320 | YEESRAQL | 0.244286 | TTTTHHHH | Homo-hexameric KaiC displays kinase, ATPase and phosphatase activities, S431 and T432 phosphorylation follows TS-pTSR-pTpS-TpS-TS |
| P20906 | 2FWN | 26 | NPGSNELP | 0.244286 | CTTTTHHH | Benzoylformate decarboxylase, incubation with benzoylphosphonate results in inhibitory Phosphorylation of active site |
| Q79PF4 | 1U9I | 432 | HISTITDT | 0.244286 | TTTTTTTT | Homo-hexameric KaiC displays kinase, ATPase and phosphatase activities; phosphorylation of T432 and S431/T426 are key components of the circadian clockwork |
| P06786 | 3L4K | 782 | AARYIYTE | 0.25 | TTTTTEEE | Topoisomerase II with DNA. Direct interaction between active site Y782 and DNA |
| Q79PF4 | 4DUG | 432 | HISTITDT | 0.25 | TCCCCCCE | Homo-hexameric KaiC displays kinase, ATPase and phosphatase activities, S431 and T432 phosphorylation follows TS-pTSR-pTpS-TpS-TS |
| Q79PF4 | 3K09 | 432 | HIDTITDT | 0.251429 | TCCCCCCE | Homo-hexameric KaiC displays kinase, ATPase and phosphatase activities, S431 and T432 phosphorylation follows TS-pTSR-pTpS-TpS-TS |
| O34952 | 2W8D | 297 | QGKTSDAE | 0.255714 | TTGGGGTT | Lipoteichoic acid synthase 2, Source of phosphate unknown; could be a small molecule and not a kinase |
| Q6P4R6 | 2QON | 701 | VTEYMENG | 0.258571 | EECCTTTE | Autophosphorylated EphA3. Confirmed by MS |
| P12931 | 1YI6 | 335 | EPIYIVTE | 0.261429 | TTTTCEEE | C’ terminal tail of Tyrosine kinase. Not in PubMed |
| Q79PF4 | 3K0A | 432 | HIATITDT | 0.27 | CCCCCCCE | Homo-hexameric KaiC displays kinase, ATPase and phosphatase activities, S431 and T432 phosphorylation follows TS-pTSR-pTpS-TpS-TS |
| P32989 | 2H7F | 274 | KRAYMATT | 0.272857 | HHHHCCHH | Viral topoisomerase bound to DNA, catalytic Y274 interacts with DNA |
| A1L1M0 | 3TNP | 139 | GRFSEPHA | 0.278571 | CCCCHHHH | cAMP-dependent protein kinase catalytic subunit alpha; T97 and T139 in catalytic subunit. It is a tetramer (Dimer of dimer ) |
| P00550 | 1VRV | 384 | IVASDAGM | 0.281429 | EEEETTTC | Exposed, phosphor-transferase system |
| P07515 | 1FU0 | 146 | NLKSIMGV | 0.282857 | ECCCHHHH | Exposed, not in pubmed, Hpr |
| P05132 | 4DH5 | 139 | GRFSEPHA | 0.284286 | CCCCHHHH | Exposed, PKA catalytic subunit with ATP and peptide inhibitor |
| P17612 | 4AE9 | 139 | GRFSEPHA | 0.288571 | CCCCHHHH | Exposed, PKA catalytic subunit, dimer |
| P00517 | 1SZM | 139 | GRFSEPHA | 0.292857 | CCCCHHHH | Exposed, PKA C-alpha with inhibitor |
| P08149 | 3EQU | 465 | FKESTARE | 0.292857 | TTTTHHHH | Exposed, penicillin binding protein 2 |
| Q9X1F5 | 1T6R | 59 | YMDSAGLG | 0.292857 | CBCCHHHH | Exposed, putative anti sigma factor antagonist |
| Q79PF4 | 3K0F | 431 | SHISAITD | 0.294286 | TTTTTTTC | Homo-hexameric KaiC displays kinase, ATPase and phosphatase activities, S431 and T432 phosphorylation follows TS-pTSR-pTpS-TpS-TS |
| P54645 | 2Y94 | 377 | ARHTLDEL | 0.294286 | TTTTTTTT | Trimeric AMPK, part of alpha –hook. not discussed in paper |
| P03076 | 1FU5 | 11 | EDLYLDIL | 0.298571 | CCCCCCCC | The N-terminal SH2 domain of the p85 subunit of PI3K, it’s a phosphopeptide |

Literature was surveyed to explain the origin and nature of the phosphor residues from eukaryotic proteins which seem relatively in accessible (<0.3 rSASA).

1.	Koutsioulis, D., Lyskowski, A., Maki, S., Guthrie, E., Feller, G., Bouriotis, V., and Heikinheimo, P. (2010) Coordination sphere of the third metal site is essential to the activity and metal selectivity of alkaline phosphatases. *Protein Sci* **19**, 75-84

2.	Wang, J., and Kantrowitz, E. R. (2006) Trapping the tetrahedral intermediate in the alkaline phosphatase reaction by substitution of the active site serine with threonine. *Protein Sci* **15**, 2395-2401

3.	Llinas, P., Masella, M., Stigbrand, T., Menez, A., Stura, E. A., and Le Du, M. H. (2006) Structural studies of human alkaline phosphatase in complex with strontium: implication for its secondary effect in bones. *Protein Sci* **15**, 1691-1700

4.	Murphy, J. E., Stec, B., Ma, L., and Kantrowitz, E. R. (1997) Trapping and visualization of a covalent enzyme-phosphate intermediate. *Nat Struct Biol* **4**, 618-622

5.	Suh, J. Y., Cai, M., Williams, D. C., Jr., and Clore, G. M. (2006) Solution structure of a post-transition state analog of the phosphotransfer reaction between the A and B cytoplasmic domains of the mannitol transporter IIMannitol of the Escherichia coli phosphotransferase system. *J Biol Chem* **281**, 8939-8949

6.	Brandt, G. S., Kneen, M. M., Petsko, G. A., Ringe, D., and McLeish, M. J. (2010) Active-site engineering of benzaldehyde lyase shows that a point mutation can confer both new reactivity and susceptibility to mechanism-based inhibition. *J Am Chem Soc* **132**, 438-439

7.	Osawa, T., Kimura, S., Terasaka, N., Inanaga, H., Suzuki, T., and Numata, T. (2011) Structural basis of tRNA agmatinylation essential for AUA codon decoding. *Nat Struct Mol Biol* **18**, 1275-1280

8.	Stroupe, M. E., Leech, H. K., Daniels, D. S., Warren, M. J., and Getzoff, E. D. (2003) CysG structure reveals tetrapyrrole-binding features and novel regulation of siroheme biosynthesis. *Nat Struct Biol* **10**, 1064-1073

9.	Iverson, T. M., Panosian, T. D., Birmingham, W. R., Nannemann, D. P., and Bachmann, B. O. (2012) Molecular differences between a mutase and a phosphatase: investigations of the activation step in Bacillus cereus phosphopentomutase. *Biochemistry* **51**, 1964-1975

10.	Wohlkonig, A., Chan, P. F., Fosberry, A. P., Homes, P., Huang, J., Kranz, M., Leydon, V. R., Miles, T. J., Pearson, N. D., Perera, R. L., Shillings, A. J., Gwynn, M. N., and Bax, B. D. (2010) Structural basis of quinolone inhibition of type IIA topoisomerases and target-mediated resistance. *Nat Struct Mol Biol* **17**, 1152-1153

11.	Schumacher, M. A., Seidel, G., Hillen, W., and Brennan, R. G. (2007) Structural mechanism for the fine-tuning of CcpA function by the small molecule effectors glucose 6-phosphate and fructose 1,6-bisphosphate. *J Mol Biol* **368**, 1042-1050

12.	Kim, C., Xuong, N. H., and Taylor, S. S. (2005) Crystal structure of a complex between the catalytic and regulatory (RIalpha) subunits of PKA. *Science* **307**, 690-696

13.	Zhang, P., Smith-Nguyen, E. V., Keshwani, M. M., Deal, M. S., Kornev, A. P., and Taylor, S. S. (2012) Structure and allostery of the PKA RIIbeta tetrameric holoenzyme. *Science* **335**, 712-716

14.	Liu, Q., Shen, Y., Liu, S., Weng, J., and Liu, J. (2011) Crystal structure of E339K mutated human glucokinase reveals changes in the ATP binding site. *FEBS Lett* **585**, 1175-1179

15.	Regni, C., Naught, L., Tipton, P. A., and Beamer, L. J. (2004) Structural basis of diverse substrate recognition by the enzyme PMM/PGM from P. aeruginosa. *Structure* **12**, 55-63

16.	Chaptal, V., Lariviere, L., Gueguen-Chaignon, V., Galinier, A., Nessler, S., and Morera, S. (2006) X-ray structure of a domain-swapped dimer of Ser46-phosphorylated Crh from Bacillus subtilis. *Proteins* **63**, 249-251

17.	Chrencik, J. E., Staker, B. L., Burgin, A. B., Pourquier, P., Pommier, Y., Stewart, L., and Redinbo, M. R. (2004) Mechanisms of camptothecin resistance by human topoisomerase I mutations. *J Mol Biol* **339**, 773-784

18.	Pattanayek, R., Williams, D. R., Rossi, G., Weigand, S., Mori, T., Johnson, C. H., Stewart, P. L., and Egli, M. (2011) Combined SAXS/EM based models of the S. elongatus post-translational circadian oscillator and its interactions with the output His-kinase SasA. *PLoS One* **6**, e23697

19.	Bera, A. K., Polovnikova, L. S., Roestamadji, J., Widlanski, T. S., Kenyon, G. L., McLeish, M. J., and Hasson, M. S. (2007) Mechanism-based inactivation of benzoylformate decarboxylase, a thiamin diphosphate-dependent enzyme. *J Am Chem Soc* **129**, 4120-4121

20.	Xu, Y., Mori, T., Pattanayek, R., Pattanayek, S., Egli, M., and Johnson, C. H. (2004) Identification of key phosphorylation sites in the circadian clock protein KaiC by crystallographic and mutagenetic analyses. *Proc Natl Acad Sci U S A* **101**, 13933-13938

21.	Schmidt, B. H., Burgin, A. B., Deweese, J. E., Osheroff, N., and Berger, J. M. (2010) A novel and unified two-metal mechanism for DNA cleavage by type II and IA topoisomerases. *Nature* **465**, 641-644

22.	Egli, M., Mori, T., Pattanayek, R., Xu, Y., Qin, X., and Johnson, C. H. (2012) Dephosphorylation of the core clock protein KaiC in the cyanobacterial KaiABC circadian oscillator proceeds via an ATP synthase mechanism. *Biochemistry* **51**, 1547-1558

23.	Schirner, K., Marles-Wright, J., Lewis, R. J., and Errington, J. (2009) Distinct and essential morphogenic functions for wall- and lipo-teichoic acids in Bacillus subtilis. *EMBO J* **28**, 830-842

24.	Davis, T. L., Walker, J. R., Loppnau, P., Butler-Cole, C., Allali-Hassani, A., and Dhe-Paganon, S. (2008) Autoregulation by the juxtamembrane region of the human ephrin receptor tyrosine kinase A3 (EphA3). *Structure* **16**, 873-884

25.	Perry, K., Hwang, Y., Bushman, F. D., and Van Duyne, G. D. (2006) Structural basis for specificity in the poxvirus topoisomerase. *Mol Cell* **23**, 343-354

26.	Suh, J. Y., Tang, C., Cai, M., and Clore, G. M. (2005) Visualization of the phosphorylated active site loop of the cytoplasmic B domain of the mannitol transporter II(Mannitol) of the Escherichia coli phosphotransferase system by NMR spectroscopy and residual dipolar couplings. *J Mol Biol* **353**, 1129-1136

27.	Kovalevsky, A. Y., Johnson, H., Hanson, B. L., Waltman, M. J., Fisher, S. Z., Taylor, S., and Langan, P. (2012) Low- and room-temperature X-ray structures of protein kinase A ternary complexes shed new light on its activity. *Acta Crystallogr D Biol Crystallogr* **68**, 854-860

28.	Hereng, T. H., Backe, P. H., Kahmann, J., Scheich, C., Bjoras, M., Skalhegg, B. S., and Rosendal, K. R. (2012) Structure and function of the human sperm-specific isoform of protein kinase A (PKA) catalytic subunit Calpha2. *J Struct Biol* **178**, 300-310

29.	Gassel, M., Breitenlechner, C. B., Konig, N., Huber, R., Engh, R. A., and Bossemeyer, D. (2004) The protein kinase C inhibitor bisindolyl maleimide 2 binds with reversed orientations to different conformations of protein kinase A. *J Biol Chem* **279**, 23679-23690

30.	Powell, A. J., Tomberg, J., Deacon, A. M., Nicholas, R. A., and Davies, C. (2009) Crystal structures of penicillin-binding protein 2 from penicillin-susceptible and -resistant strains of Neisseria gonorrhoeae reveal an unexpectedly subtle mechanism for antibiotic resistance. *J Biol Chem* **284**, 1202-1212

31.	Etezady-Esfarjani, T., Placzek, W. J., Herrmann, T., and Wuthrich, K. (2006) Solution structures of the putative anti-sigma-factor antagonist TM1442 from Thermotoga maritima in the free and phosphorylated states. *Magn Reson Chem* **44 Spec No**, S61-70

32.	Xiao, B., Sanders, M. J., Underwood, E., Heath, R., Mayer, F. V., Carmena, D., Jing, C., Walker, P. A., Eccleston, J. F., Haire, L. F., Saiu, P., Howell, S. A., Aasland, R., Martin, S. R., Carling, D., and Gamblin, S. J. (2011) Structure of mammalian AMPK and its regulation by ADP. *Nature* **472**, 230-233

33.	Weber, T., Schaffhausen, B., Liu, Y., and Gunther, U. L. (2000) NMR structure of the N-SH2 of the p85 subunit of phosphoinositide 3-kinase complexed to a doubly phosphorylated peptide reveals a second phosphotyrosine binding site. *Biochemistry* **39**, 15860-15869
