# Supplementary material for: Structural interrogation of phosphoproteome identified by mass spectrometry reveals allowed and disallowed regions of phosphoconformation
Source: BMC Struct Biol. 2014 Mar 11;14:9. doi: 10.1186/1472-6807-14-9 (PMC4007652; doi:10.1186/1472-6807-14-9)
Supplement: Additional file 1: Figure S1 — Schema/work flow of analysis of Phosphosites. Figure S2. Accessibility of Phosphosites before and after energy minimization of the corresponding protein structures. Figure S3. Physico chemical properties of amino acids within the posphosites in allowed and disallowed region of conformation. Figure S4. Structure of Phosphosites within the ambiguous dataset. Table S1. Accessibility of Phosphosites from the PhosphoSitePlus Data base. Table S2. Accessibility of octapeptides carrying the phosphorylated residue in eukaryotic and prokaryotic proteins within the PDB database. Table S3. Nature and origin of Phosphosites (S/T/Y) with rSASA values less than 0.3 within the PDB data set of eukaryotic proteins. Table S4. Functional Classification of Phosphosites. Table S5. Extent of accessibility of phosphosites with different levels of curation. Table S6. Statistics for the Cα-Elastic Network Model Normal Mode Analyses of selected proteins. Table S7. DEPTH values an alternate measure of accessibility of phosphosites. Table S8. Accessibility as a measure of confidence in the identification of phosphosites. [file 1472-6807-14-9-S1.zip › 1695290998113254_TableS5.doc]

| **rSASA** | **Pubmed_LTP/MS(%)** | **CST_MS (%)** | **Both** |
| --- | --- | --- | --- |
| <0.2 (Disallowed) | 261 (13%) | 485 (16.8%) | 130 |
| >0.2  (Allowed) | 1752 (87%) | 2394 (83.2%) | 925 |

**Supplementary data Table 5:** Extent of accessibility of phosphosites with different levels of curation.

PhosphoSitePlus is a depository of both high and less well curated phosphorylation sites (CSA MS) from human and mouse . To rule out the possibility that inaccessibility could be a reflection of level of curation, we segregated this data into HTP MS with Pubmed reference only, CSA MS data only and those with records from both data set and classified them on the conformational map.
