# Supplementary material for: Structural interrogation of phosphoproteome identified by mass spectrometry reveals allowed and disallowed regions of phosphoconformation
Source: BMC Struct Biol. 2014 Mar 11;14:9. doi: 10.1186/1472-6807-14-9 (PMC4007652; doi:10.1186/1472-6807-14-9)
Supplement: Additional file 1: Figure S1 — Schema/work flow of analysis of Phosphosites. Figure S2. Accessibility of Phosphosites before and after energy minimization of the corresponding protein structures. Figure S3. Physico chemical properties of amino acids within the posphosites in allowed and disallowed region of conformation. Figure S4. Structure of Phosphosites within the ambiguous dataset. Table S1. Accessibility of Phosphosites from the PhosphoSitePlus Data base. Table S2. Accessibility of octapeptides carrying the phosphorylated residue in eukaryotic and prokaryotic proteins within the PDB database. Table S3. Nature and origin of Phosphosites (S/T/Y) with rSASA values less than 0.3 within the PDB data set of eukaryotic proteins. Table S4. Functional Classification of Phosphosites. Table S5. Extent of accessibility of phosphosites with different levels of curation. Table S6. Statistics for the Cα-Elastic Network Model Normal Mode Analyses of selected proteins. Table S7. DEPTH values an alternate measure of accessibility of phosphosites. Table S8. Accessibility as a measure of confidence in the identification of phosphosites. [file 1472-6807-14-9-S1.zip › 1695290998113254_TableS8.doc]

**Supplemental Table 8** Accessibility as a measure of confidence in the identification of phosphosites

| **Peptide Sequence** | **Score** | **Protein** | **Uniprot ID**  **(Human)** | **Octapeptide** | **PDB** | **Sequence**  **coverage** | **Chain and position** | **SASA** | **rSASA** |
| --- | --- | --- | --- | --- | --- | --- | --- | --- | --- |
| T*TTTAVISK | 0 | DIPHOSPHOMEVALONATE DECARBOXYLASE. | MVD1 | QLKTTTTA | 3D4J | 100 | A-47 | 224.16 | 0.158 |
| TLNMTTS*PEEK | 0 | GMP SYNTHASE. | GUAA | MTTSPEEK | 2VXO | 97.2583 | A-329 | 364.97 | 0.249 |
| YGMGTS*VER | 0 | PYRUVATE DEHYDROGENASE E1 COMPONENT ALPHA SUBUNIT, SOMATIC FORM,MITOCHONDRIAL PRECURSOR. | ODPA | MGTSVERA | 2OZL | 92.5641 | A-200 | 539.57 | 0.349 |
| S*QSAAVT*PSSTTSSTR | 9.34 | ADHESION REGULATING MOLECULE 1 PRECURSOR. | ADRM1 | SSRSQSAA | 2KR0 | 100 | A-208 | 1122.69 | 0.819 |
| SPSEESAPTTS*PESVSGSVPSSGSSGR | 12.2 | UV EXCISION REPAIR PROTEIN RAD23 HOMOLOG A. | RD23A | PTTSPESV | 1OQY | 99.7245 | A-130 | 1035.67 | 0.804 |
| GVLFGVPGAFT*PGCSK | 14.52 | PEROXIREDOXIN 5, MITOCHONDRIAL PRECURSOR. | PRDX5 | GAFTPGCS | 2VL9 | 75.2336 | A-41 | 500.15 | 0.41 |
| EFAGEDTS*DLFLEER | 16.22 | EXPORTIN-1. | XPO1 | EDTSDLFL | 3GB8 | 100 | A-1028 | 520.23 | 0.40 |
| LDIDS*PPITAR | 18.34 | PYRUVATE KINASE 3 ISOFORM 1 VARIANT. | KPYM | DIDSPPIT | 1ZJH | 99.4350 | A-33 | 739.68 | 0.587 |

A very high quality data set where scores were provided for strength or ambiguity in MS identification was chosen as a test case for using accessibility as an index to increase the confidence of identification. An ‘A’ score of <19 indicated ambiguity but >19 indicated high confident assignment. rSASA values for those phosphosites with a match in PDB were calculated. 7 out of eight ambiguous sites are likely to be phosphorylated.
